# Arrhythmic Risk Stratification and Sudden Cardiac Death Prevention in Duchenne Muscular Dystrophy: A Critical Appraisal

**DOI:** 10.31083/RCM27089

**Published:** 2025-03-11

**Authors:** Domenico D’Amario, Alessandra Arcudi, Maria Lucia Narducci, Valeria Novelli, Francesco Canonico, Alessandro Parodi, Gabriele Dell’Era, Marco Di Francesco, Renzo Laborante, Josip Andelo Borovac, Mattia Galli, Eugenio Maria Mercuri, Giuseppe Vergaro, Antonio Dello Russo, Anthea Tonia D’Amico, Antonio Bisignani, Rachele Adorisio, Giulio Pompilio, Giuseppe Patti

**Affiliations:** ^1^Department of Translational Medicine, University of Eastern Piedmont, 28100 Novara, Italy; ^2^Thoraco-Cardio-Vascular Department, Azienda Ospedaliero-Universitaria Maggiore della Carità, 28100 Novara, Italy; ^3^Department of Cardiovascular Science, Fondazione Policlinico Agostino Gemelli IRCCS, 00168 Rome, Italy; ^4^Department of Cardiovascular Science, Catholic University of the Sacred Heart, 00168 Rome, Italy; ^5^Department of Cardiac Surgery, Centro Cardiologico Monzino-IRCCS, 20138 Milan, Italy; ^6^Clinic for Heart and Vascular Diseases, University Hospital of Split, 21000 Split, Croatia; ^7^Maria Cecilia Hospital, GVM Care and Research, 48033 Cotignola, Italy; ^8^Fondazione Toscana G. Monasterio, 56126 Pisa, Italy; ^9^Cardiology and Arrhythmology Clinic, University Hospital “Ospedali Riuniti Umberto I-Lancisi-Salesi”, 60126 Ancona, Italy; ^10^Center of Excellence in Cardiovascular Sciences, Ospedale Isola Tiberina-Gemelli Isola, 00153 Rome, Italy; ^11^Advanced Cardiovascular Therapy Unit, Bambino Gesù Pediatric Hospital and Research Institute, 00165, Rome, Italy

**Keywords:** Duchenne muscular dystrophy, dilated cardiomyopathy, sudden cardiac death prevention, arrhythmic risk stratification, implantable cardioverter defibrillator

## Abstract

Duchenne muscular dystrophy (DMD) is a genetic progressive neuromuscular disorder characterized by early-onset proximal muscle weakness and significant long-term pulmonary and cardiac involvement. Due to the early pharmacological treatments and the wider adoption of non-invasive ventilation, life expectancy has significantly increased in recent years, highlighting the relevance of DMD-related cardiomyopathy and fatal arrhythmias, especially in the late stage of the disease. Current guideline-derived evaluation of sudden cardiac death (SCD) in DMD lacks accuracy, leading to inadequate arrhythmic risk stratification and jeopardized SCD prevention strategies. This review aims to outline these critical issues, proposing an integrative approach encompassing manifold tools such as an imaging-derived systematic and comprehensive evaluation (speckle-tracking echocardiography and magnetic resonance imaging), the electrophysiological study, the 3-dimensional electroanatomic mapping, and a multidimensional clinical examination. This approach might lead to more personalized management along with an effective arrhythmia-prevention strategy aiming to balance clinical care goals, patient expectations, and ethical considerations.

## 1. Introduction

Duchenne muscular dystrophy (DMD) is an X-linked recessive disorder caused by mutations or deletions in the Dystrophin gene resulting in an absent or nonfunctional protein [1]. Dystrophin connects the cytoskeleton to the extracellular matrix thus being essential to protect the muscular cell from mechanical strain-induced damage [2]. Without dystrophin, muscular fiber membrane integrity is compromised and a fibro-fatty replacement in skeletal and cardiac muscle is induced, leading to cell death and muscle-wasting clinical manifestations [1]. DMD is characterized by progressive muscular strength and mass reduction, resulting in a patient’s loss of independent gait by the age of 10, respiratory dysfunction by the age of 20, and cardiopulmonary failure and death between ages 20 and 40 [3]. Recent advancements in the clinical management of the disease, as the early pharmacological treatment that mitigates the devastating consequences of skeletal muscle dysfunction and the concomitant use of non-invasive ventilation, have determined an improvement in DMD patients’ life quality and length [4]. In this regard, heart failure (HF) and sudden cardiac death (SCD) come to prominence as the leading causes of death [3, 5, 6]. DMD-related cardiomyopathy may present as Non- Dilated Left Ventricular Cardiomyopathy (LVNDC) or as Dilated Cardiomyopathy (DCM) as reported by the last European society of Cardiology (ESC) guidelines over cardiomyopathies. DMD-cardiomyopathy is not a classic DCM form, with severe ventricular dilation that appears only in the last stages, however, to assure consistency with ESC nomenclature [7], we’ll refer to DMD-related cardiomyopathy as DCM.

**Fig. 1.**
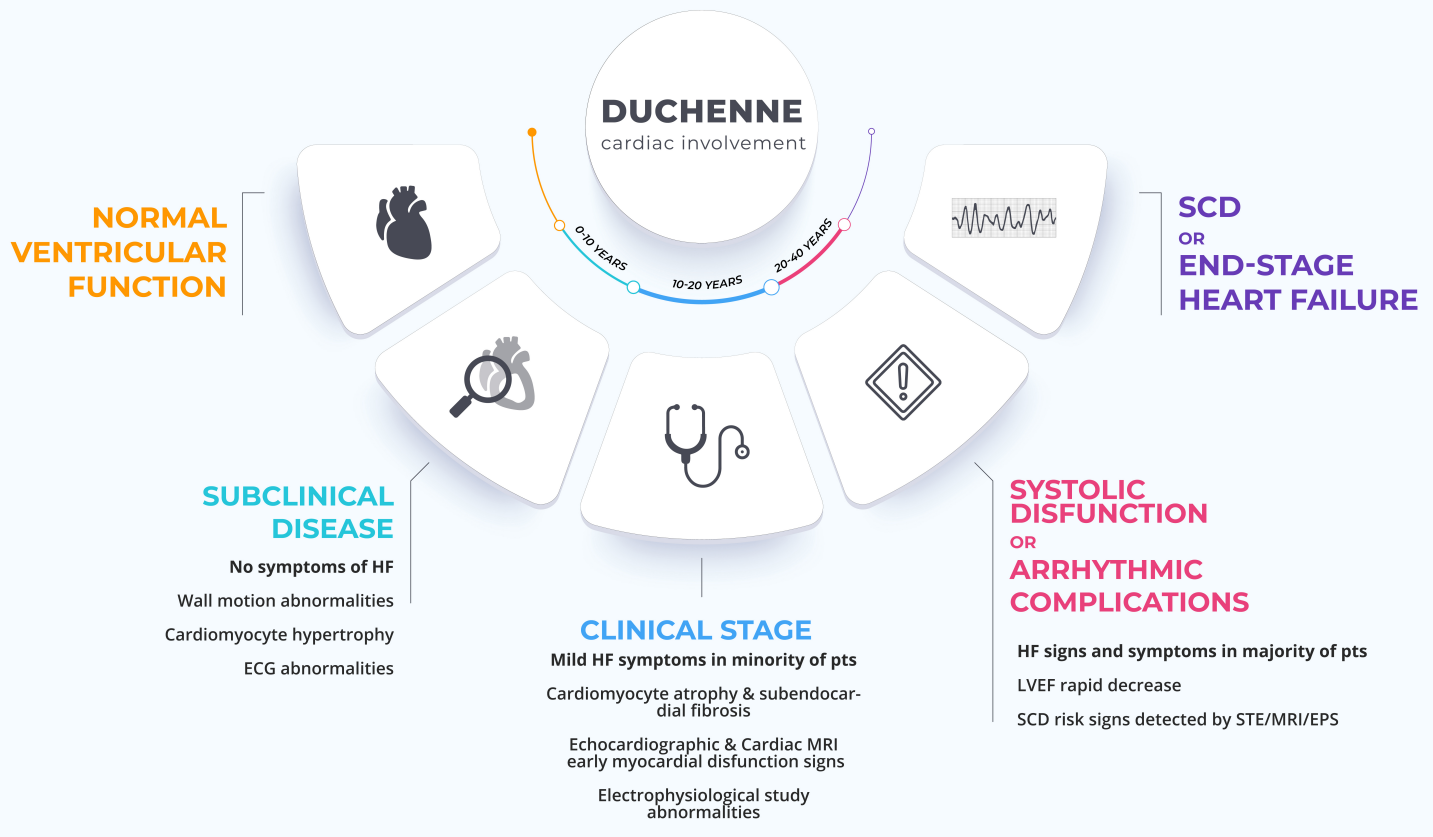
**DMD cardiac involvement history**. The figure shows the cardiac involvement in the patient with DMD, from normal ventricular function to subclinical disease, arrhythmic complications or systolic dysfunction, up to SCD or end-stage HF (Some graphic elements used are under free license from freepik.com). DMD, Duchenne muscular dystrophy; SCD, sudden cardiac death; HF, heart failure; ECG, electrocardiogram; LVEF, left ventricular ejection fraction; EPS, electrophysiological study; pts, patients; MRI, magnetic resonance imaging; STE, speckle tracking echocardiography.

Despite a predictable cardiac involvement, these patients often face delay in cardiac evaluation with a belated cardiologist referral mainly because of a long preclinical phase preceding the onset of clinically detectable cardiomyopathy [3] (Fig. 1). Skeletal muscles, bones and respiratory system represent the most common non-cardiac involvement in DMD, and they play a major role in masking cardiovascular symptoms, thus hindering HF symptom recognition [3].

In this evolving scenario, the pathophysiological mechanisms and prevalence of arrhythmias are still debated, especially during the progression of the disease. Consequently, a shared strategy aiming to prevent SCD is lacking. Recommendations for an implantable cardioverter-defibrillator (ICD) in DMD patients have traditionally been extrapolated from adult HF guidelines, with left ventricular ejection fraction (LVEF) as the main element to be considered. However, using a single, dynamic, and operator-dependent parameter (i.e., LVEF) as a long-term SCD predictor may carry relevant limitations, as recently high-lightened [8, 9]. The occurrence of warning signs preceding SCD, and the development of dynamic multidimensional analysis hold great potential to herald a new paradigm in short and long-term prevention of SCD in this cardiology area.

This review aims to summarize current knowledge on arrhythmic risk stratification in DMD and to propose possible strategies for SCD prevention, providing a complementary, multidimensional approach, carefully balancing clinical care priorities together with realistic and shared expectations coming from patients and their families/caregivers.

## 2. Pathophysiological Basis of Arrhythmic Disorders in DMD

**Fig. 2.**
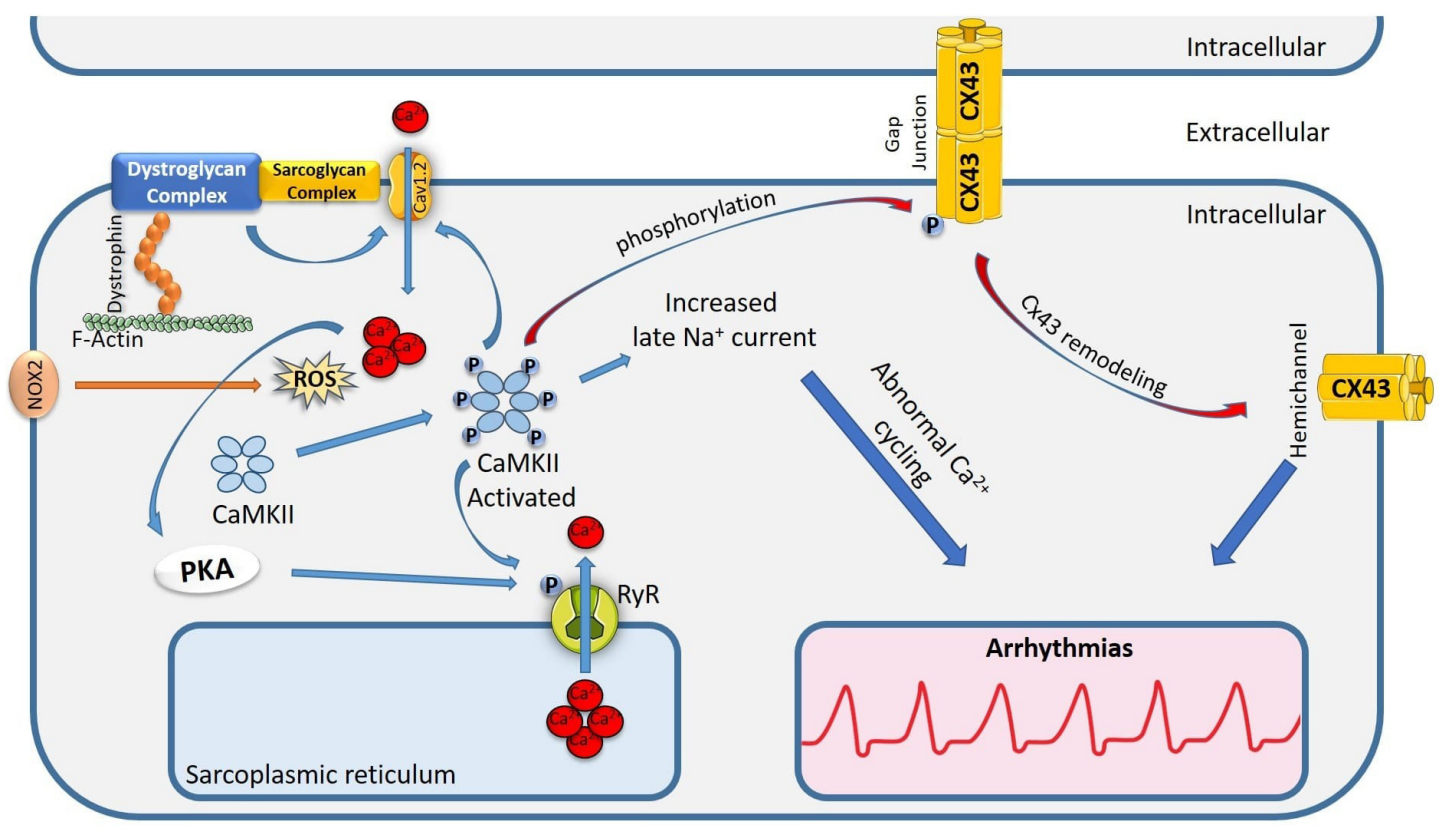
**Schematic representation of underlying mechanisms of arrhythmias in DMD patients**. The figure shows the dystrophic pathways involving the lateralization of Cx43, leading to improper gap junction formation, Na^+^/Ca^2+^ deregulation, and RyR phosphorylation. RyR, ryanodine receptor; Cx43, connexins 43; Cav1.2, Ca^2+^ channel voltage-dependent 1.2.; NOX2, NADPH oxidase 2; PKA, protein kinase A; CaMKII, Ca^2+^/calmodulin-dependent protein kinase II; ROS, reactive oxygen species; DMD, Duchenne muscular dystrophy.

The pathogenesis of arrhythmic disorders in DMD remains controversial [10] with both electrical and structural alterations having been proposed to explain this feature of the disease. In cardiomyocytes, electrical conduction depends on gap junctions, composed of two-faced hemichannels, made up of six units of connexins (Cx). Cx43 is the most expressed connexin in cardiomyocytes and it is the main responsible protein for the propagation of action potential [11] (Fig. 2). *Mdx* mice are a useful model to study DMD-mutated gene population with non-functional dystrophin. In *Mdx* mice cardiomyocytes, Cx43 is lateralized outside the limits of the gap junction, creating additional pores with increased permeability leading to an augmented arrhythmic risk [11]. Furthermore, *Mdx* mice cardiomyocytes are characterized by chronic phosphorylation and nitrosation of the ryanodine receptor (RyR) 2, thus enhancing the amount of calcium ion Ca^2+^ in the cytoplasm, triggering an increased risk of ventricular arrhythmias (VA) [12]. Abnormalities in voltage-dependent sarcolemmal channels may also play a role in dystrophic hearts. In a dystrophin deficient murine model, dystrophin regulation of Ca^2+^ channel voltage-dependent 1.2 (Cav1.2) seems to be lost and a L-type Ca^2+^channel *gain-of-function* has been related to atrioventricular nodal conduction and ventricular repolarization abnormalities [13]. Moreover, the reduced sodium ion (Na^+^) currents (INa) in Purkinje fibers of mdx mice affecting ventricular conduction may contribute to ventricular asynchrony and re-entrant arrhythmias [14]. Further evidence of protein interaction in VA genesis comes from a humanized mdx (hDMDdel52-null) mouse model. Ventricular activation and repolarization defects anticipate structural abnormalities in this lack-of-dystrophin model, these functional alterations were otherwise absent in hDMDdel52-low mice, demonstrating that low dystrophin levels are sufficient to prevent early electrical modifications and subsequent structural cardiac remodeling [15]. Indeed, arrhythmogenesis is strongly related to the heart structural disarrangement deriving from protein impairment. In DMD, a direct correlation between progressive fibrosis extension and a higher incidence of non-sustained/sustained ventricular tachycardias (VTs), together with a longer QT dispersion (QTd) has been demonstrated [16].

In summary, the pathophysiology of electrical instability in DMD is complex and still poorly understood. Its biomolecular mechanisms vary throughout the different stages of the disease and mainly involve gap junctions, voltage channels, and phosphorylation/nitrosation of RyR leading to myocardial scar progression.

## 3. Arrhythmic Disorders in DMD

Sinus tachycardia is the most frequent electrocardiogram (ECG) presentation in DMD patients; it occurs early in the natural history of the disease, even with poor mobility, and it is probably related to an autonomic dysfunction mainly involving a parasympathetic system dysregulation [17, 18]. Premature atrial contractions (PACs) are common and tend to increase with the progression of cardiac involvement. Ectopic atrial tachycardia (EAT) and atrial fibrillation/atrial flutter (AF/AFL) have been found in DMD patients, especially in those with DCM and respiratory dysfunction [17, 19]. However, no direct correlation between atrial arrhythmias and SCD has yet been demonstrated. VAs, especially as ventricular premature beats (VPB) may occur in up to 30% of DMD patients [20], increasing along with disease progression, additionally they have been documented in DMD patients suffering SCD [19]. A fragmented QRS (fQRS) may be a marker of cardiac involvement in DMD patients, moreover, an association between fQRS, left ventricular (LV) fibrosis extent, LV dysfunction, and VAs burden seems likely [21]. In addition, QTd has also been identified as an independent risk factor for VAs occurrence [19]. Atrial and ventricular arrhythmias are more common in DMD patients with reduced LVEF (<35%), but interestingly it does not seem to predict sudden cardiac death or life-threatening arrhythmia onset [22].

Other ECG features such as atrioventricular blocks (AVB) are uncommon during DMD course, while short PR intervals can be found in these patients [19]. Attention should be paid to QRS duration instead, since it tends to progressively increase with age and may indicate a higher risk of conduction disorders, irrespective of LV systolic function [23]. However, no disease-specific ECG sign is present in DMD, arising uncertainties in clinical practice. To date, due to lack of evidence, no predictor of arrhythmic events can be used to identify high risk patients.

## 4. Arrhythmic Risk Stratification and SCD Risk Prevention: Are Current Guidelines Enough?

### 4.1 Pharmacological Therapy

Current approaches to arrhythmic risk definition and SCD prevention are mainly based on ESC guidelines where no specific DMD management is reported. Additionally, no differentiation from DMD-related cardiomyopathy to any other form of non-ischemic dilated cardiomyopathy is stated (Class I, Level C) [7, 8]. Therefore, in terms of SCD prevention and anti-remodeling therapies, DMD cardiomyopathy treatment mainly relies on angiotensin-converting enzyme inhibitors (ACEI)/angiotensin receptor blockers (ARB), mineralocorticoid receptor antagonists (MRA), beta-blockers (BB), while angiotensin receptor-neprilysin inhibitors (ARNI) and sodium-glucose co-transporter-2 (SGLT2) inhibitors have recently been added [7, 24].

According to the National Heart, Lung, and Blood Institute (NHLBI)/Parent Project Muscular Dystrophy (PPMD) Working Group, ACEIs or ARBs are the first-line treatment for cardiac involvement in DMD both in symptomatic and asymptomatic LV systolic dysfunction, regardless of age. Furthermore, their use is recommended even before reduced-ejection fraction (EF) detection in patients aged ≥10, to delay heart remodeling onset and progression [25, 26]. BBs are only recommended when LV dysfunction is evident, with a greater magnitude of efficacy in asymptomatic patients [25]. With regards to MRA, they are approved in DMD heart involvement irrespective of LV systolic function with data showing effects on myocardial fibrosis and circumferential LV strain using cardiac magnetic resonance (CMR) [27]. The role of ARNI or SGLT2 in preventing DMD cardiomyopathy development and progression has not been extensively studied [28].

Corticosteroids, a cornerstone of DMD therapy, seem to have a beneficial effect enhancing cardiac protection; a longer steroid treatment duration has indeed been associated with a lower age-related presence of late gadolinium enhancement (LGE) [29]. With regards to Ivabradine, which inhibits the hyperpolarization-activated cyclic nucleotide-gated channel (HCN4 channel), it seems to be promising as it was shown to ameliorate two important targets in DMD-related cardiomyopathy: heart rate control and LVEF preservation. However, these studies dealt with end stage DMD cardiomyopathy and they are mainly retrospective [30, 31], thus more evidence is needed.

Standard antiarrhythmic drugs (AADs) are approved by the NHLBI/PPMD Working Group in secondary prevention, when indicated. Therefore, in patients with evidence of VA, medical therapy optimization, such as combining amiodarone with BBs or their replacement with sotalol should be evaluated [8]. Catheter ablation should be considered when AADs are not effective or tolerated (ESC guidelines, Class IIa, level of evidence C), while it is recommended when a symptomatic bundle branch re-entrant tachycardia is present (Class I, level of evidence C) [8]. However, no specific studies on both supraventricular and ventricular arrhythmias management in DMD can be found in the literature.

The occurrence of AFL and AF has been reported in DMD, but studies addressed to specifically evaluate hemorrhagic and thromboembolic risk in this population are lacking. CHA2DS2-VASc and HAS-BLED scores have been validated in older patients whose predisposing factors and comorbidities strongly differ from DMD patients [32]. Consequently, there is an urgent need to develop validated specific scores in this subset of patients. Lastly, considering the novel gene therapies as promising opportunities for DMD, some advances should be reported. A size reduction in CMR-detected scar has been demonstrated in DMD patients treated with an intracoronary allogeneic cardiac progenitor cell population, known as cardio-sphere-derived cells (CDC, CAP-1002), suggesting a possible protective role on the occurrence of life-threatening arrhythmias. However, most of these studies are at a preclinical or clinical-phase I/II stage highlighting the need to build stronger evidence, to make them an effective option for DMD experiencing VAs [33].

### 4.2 Device Therapy 

The AHA/ACC/HRS and the ESC guidelines recommend ICD implantation in DCM in primary prevention, in patients with NYHA class II-III and EF <35%, despite optimal pharmacological therapy (OPT) (Class I, level of evidence A and Class IIa, level of evidence A, respectively) [8, 24, 34]. In secondary prevention, implantation is recommended as a Class I recommendation both in AHA and ESC guidelines [8, 24, 34].

Nowadays, the traditional LVEF-centered evaluation for ICD implantation in primary prevention is being unhinged by the introduction of new stratification tools in DCM. The poor prognostic value of EF in discriminating SCD risk has been recently highlighted by the most recent SCD guidelines which consider other parameters such as LGE on CMR, programmed electrical stimulation inducibility, sustained monomorphic ventricular tachycardia (SMVT), syncope history, and genetic testing, to be more reliable in guiding the implantation choice [8].

Even if a step towards a more systematic and personalized model of arrhythmic risk stratification in DCMs has been made, a specific algorithm for DMD which integrates clinical, practical, and ethical considerations, is still lacking. Of note, guidelines discourage the use of ICDs in patients with a life expectancy <1 year, consequently DMD patients have been largely excluded from clinical trials that assessed the benefits of ICD implantation. Moreover, a predominant part of the neuromuscular disorders (NMDs) population is paediatric, thus raising doubts on the correct management, since most of the evidence pertaining to implantable devices comes from adults.

In DMD patients, a significant association between sustained arrhythmias/SCD occurrence and LVEF decrease has not been shown [22] and several studies reported a possible misclassification of high-risk patients when only conventional echocardiographic parameters, such as LVEF, are considered [35]. Therefore, to identify the best candidate and the appropriate timing to implant ICDs in DMD patients is not an easy task.

In patients with DMD, the risk/benefit ratio assessment associated with procedural aspects of the implantation is a requiring task for clinicians [36]: the combination of respiratory failure with a restrictive pattern, severe kyphoscoliosis, and muscle weakness increases the complexity of implantation, with a rising occurrence of access-site related and sedation-related complications.

The association of disease-related reduced mobility and venous stasis after implantation additionally increases the risk of pocket infection, venous obstruction and/or thrombo-embolism [32, 36]. Several pediatric ICD studies have also demonstrated a higher rate of ICD inappropriate shocks when compared to adults [37]. They are painful events for patients, grievous for the relatives and caregivers and they are associated with worsened psychosocial and clinical outcomes.

Recently, a further attempt in personalizing NMD patient care with cardiac involvement has been made by a HRS expert consensus. It provides the most up-to-date recommendation about diagnosis and management of arrhythmic complications in each NMD. Nevertheless, the level of evidence and/or class of recommendation have been downgraded compared to prior guidelines to reflect the underrepresentation of NMDs patients in clinical studies. With regard to ICD implantation indication in DMD patients for primary prevention, the consensus established a IIa class recommendation for those with EF <35% omitting HF status (i.e., NYHA class) for the first time, due to the low reliability of functional evaluation in these patients [32]. Moreover, the 2022 ESC guidelines on SCD introduced the presence of significant LGE on CMR as an indication to ICD implantation in DMD patients (Class IIb, level of evidence C) [8]. Nevertheless, the recent 2023 ESC guidelines on cardiomyopathies included DMD in the larger group of DCMs, without giving specific and integrated indications for the stratification of the arrhythmic risk [7].

DMD patients present a low risk of clinically relevant bradyarrhythmia while their prognosis does not differ from that observed in the general population. Therefore, specific-disease recommendations do not differ from traditional pacing indications [32]. However, increased peri- and intra-procedural risk of complications should be considered, especially when the treatment is addressed to asymptomatic or minimally symptomatic individuals [32]. To date, only case reports and case series have evaluated the clinical benefit and outcome of pacemakers in DMD patients, thus clinical studies enrolling this cohort of patients are compelling [32].

Device therapy is not limited to ICDs, but it may involve cardiac resynchronization therapy (CRT) in the case of wide QRS and reduced LVEF [24]. In a DMD patient where an upgrade from a dual-chamber to biventricular pacing was performed, stabilization of LV systolic function, regression of inter- and intra-ventricular asynchrony and a decrease in systolic pulmonary artery pressure have been shown at the 1 month-follow-up [38]. In the same patient, at the 5 year follow-up, LV systolic function improvement and LV end-diastolic diameter reduction was reported [38]. Further studies aiming to define patients that may benefit from CRT (both CRT pacemaker-P and CRT defibrillator-D devices) in terms of LVEF improvement and symptom reduction (dominantly dyspnea) should be proposed, notably because of the high prevalence of wide QRS complex and extensive fibrosis associated to dystrophinopathy cardiomyopathy, two well-known predictors of poor response to CRT treatment. Lastly, some evidence has suggested that the use of a left ventricular assist device as destination therapy, in very selected cases of DMD-related DCM, could be an option as a palliative approach when no other therapeutic options are present [39].

In summary, the assessment and the consequent management of arrhythmias in DMD patients still represent an unresolved question, majorly because of the scarcity of clinical studies, the many limitations to device therapy, and the resulting weakness of evidence (Table 1, Ref. [22, 33, 36, 40, 41, 42, 43, 44]) [27].

**Table 1.** **List of clinical studies specifically evaluating the occurrence of arrhythmias or the use of ICD and CRT in patients with DMD**.

Author, Year	Sample size, (Duchenne %)	Mean age (years)	Treatment	DMD-CM severity	Study design	Outcome variable	Fup (months)	Result
Ogata *et al*., 2009 [42]	52 (100%)	19.5	ACEI + BB in symptomatic and asymptomatic patients with LV dysfunction	LVEF ≤45%	Retrospective case series	All-cause mortality	120	ACEI + BB had a beneficial effect on long-term survival of DMD and HF patients.
Matsumura *et al*., 2010 [41]	54 (100%)	21.3	Carvedilol vs placebo	LVEF <50%	Open label cohort study	All-cause mortality; deterioration of HF; severe arrhythmia	60	Survival free from death, deterioration of HF and arrhythmia was significantly higher in the BB group. No difference in LVEF and BNP. Severe arrhythmic events were 0 and 1 in the BB vs no-BB groups.
Villa* et al*., 2015 [22]	235 (100%)	13	Holter monitoring	Three groups (EF ≥55%, 35–54% and <35%)	Retrospective cohort study	Holter monitoring SCD prediction power	60	VAs more frequent in EF <35% group, but no SCD events or ICD implantation in any group.
Raman* et al*., 2017 [40]	11 (100%)	13	Eplerenone vs placebo	LVEF ≥45%; LV fibrosis	Prospective observational (extension of Raman 2015)	Parameters of LV function, blood biomarkers, AE and hospital admissions due to HF, arrhythmias, death, hyperkalaemia	24	Reduced the 12-month decline in LV systolic function compared to placebo. No biomarkers changed. No episodes of hospitalization, arrhythmia, HF, or death.
Taylor* et al*., 2019 [33]	25 (100%)	17.8	CAP-1002 vs control	Cardiomyopathy with fibrosis in ≥4 LV segments	Phase I/II, randomized, controlled, open-label trial	Reduction in Thrombolysis in MI grade flow >3 minutes; sudden unexpected death; MACE	12	Intracoronary CAP-1002 in DMD appears safe and demonstrates signals of efficacy on both cardiac and upper limb function.
Wittlieb-Weber* et al*., 2019 [44]	436 (100%)	14.9	ICD	ICD use was NSVT +FS ≤17% or EF <30% or FS <16%	Retrospective cohort study	ICD utilization and efficacy in patients with DMD	120	A greater percentage of subjects with severe LV systolic dysfunction who were alive at study end had an ICD implanted. 2 patients received 2 appropriate shocks for VT. No subject had an inappropriate shock or lead infection. One subject had a lead fracture.
Palladino* et al*., 2019 [36]	18 (28%)	21.8	ICD and CRT-D	ICD and CRT-D use in patients with subjective symptoms, EF ≤35% or in presence of arrhythmias	Retrospective cohort study	ICD and CRT-D utilization and efficacy in patients with dystrophinopathic cardiomyopathy	19.2	67% of patients received an ICD, while 33% received CRT-D. 25% of patients referred an improvement in cardiac symptoms and daily life activities. Only 1 patient had implant-related complications.
McCulloch *et al*., 2020 [43]	9 (100%)	20.4	ICD	EF<35	Retrospective cohort study	ICD in asymptomatic patients with severe LV dysfunction receiving guideline-guided medical therapy	36.1	ICD implantations were associated with two appropriate shocks for ventricular tachycardia in two patients, no inappropriate shocks, and one lead fracture.

Abbreviations: Fup, follow up; ACEI, angiotensin converting enzyme inhibitor; AE, adverse events; BB, beta-blocker; HF, heart failure; ICD, implantable cardioverter defibrillator; LV, left ventricular; LVEF, left ventricular ejection fraction; NSVT, non-sustained ventricular tachycardia; RCT, randomized controlled trial; FS, fractional shortening; VT, ventricular tachycardia; VAs, ventricular arrhythmias; SCD, sudden cardiac death; CRT, cardiac resynchronization therapy; DMD-CM, Duchenne muscular dystrophy-cardiomyopathy; MACE, major adverse cardiovascular event; EF, Ejection Fraction; CRT-D, cardiac resynchronization therapy with defibrillator; BNP, brain natriuretic peptide; MI, myocardial infarction.

## 5. Arrhythmic Risk Stratification and SCD Risk Prevention: Novel Perspectives and Proposals

### 5.1 Speckle-Tracking Echocardiography

Echocardiography is considered the first-line imaging technique in non-ischemic DCM assessment, providing several prognostic indicators, such as left ventricular systolic and diastolic function and right ventricular performance. Nevertheless, the role of each of the above parameters in DCM arrhythmic risk stratification is still debated. Recently, a useful tool has been integrated in DCM evaluation: global longitudinal strain (GLS). This has been shown to have the ability, analyzing mechanical dispersion (a mechanical dyssynchrony surrogate), to predict sustained VA or SCD in these patients [45]. In addition, GLS showed a good correlation with more sophisticated imaging techniques like CMR and especially with LGE, a well-known marker of fibrosis whose presence increases arrhythmic risk [46]. Limitations about the application of GLS in DMD should be undoubtedly recognized, as thoracic deformations and ventilators used to treat respiratory failure may lessen the overall echocardiographic quality, being high echo quality a *conditio sine qua non*, for GLS application. However, GLS is emerging as a feasible promising technique for early detection of LV myocardial dysfunction in the DMD, as recently shown in a pediatric population with normal LVEF and no overt HF symptoms [47]. This amount of evidence about GLS suggests its usefulness as an integrated tool for early detection of heart involvement in DMD and arrhythmic risk evaluation, thus giving it a place in the multiparametric approach for an ICD implantation decision, together with other markers, such as LGE in CMR.

### 5.2 Cardiac Magnetic Resonance Imaging

Localization and extension of myocardial fibrosis assessed by LGE in CMR is an attractive and emerging element in the evaluation of arrhythmic risk in DCM [9]. Interestingly, its prognostic value for SCD risk stratification is significant, related to scar extent and is independent from LV function [9]. Recommendations for noninvasive cardiac imaging state that in patients with VAs in which a structural heart disease is suspected, CMR can be useful for detecting and characterizing the underlying cardiac condition [34]. On the other hand, in cardiomyopathy patients, CMR should be considered to improve risk stratification and management [7].

**Fig. 3.**
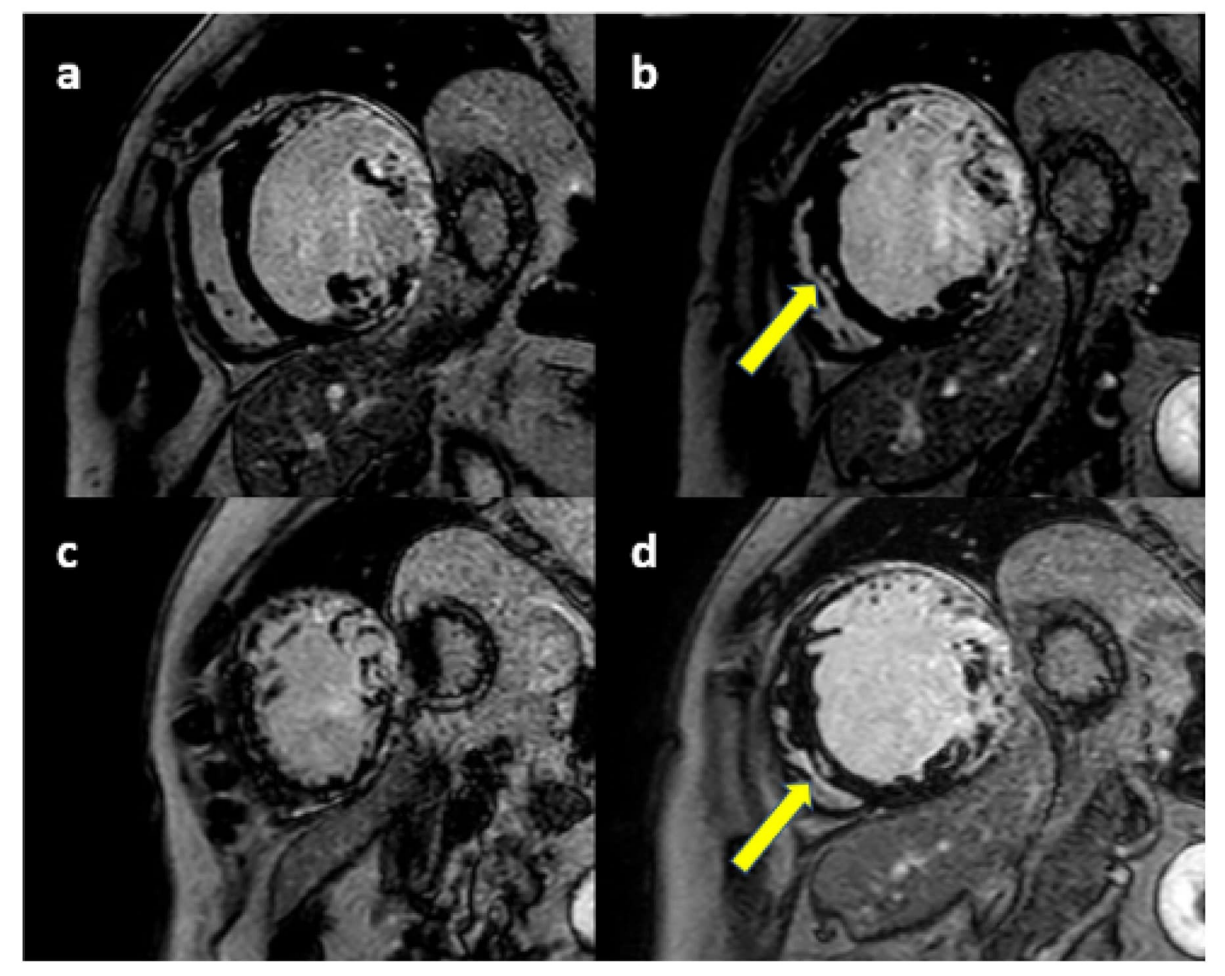
**CMR of a DMD patient**. Short Axis LGE images showing extensive myocardial fibrosis of the lateral wall of the left ventricle, reaching the anterolateral and inferolateral walls: basal (a) midventricular (b) apical (c). Epicardial and mid-wall fibrosis is also presente in the interventricular septum (arrows in b and d). CMR, cardiac magnetic resonance; LGE late gadolinium enhancement; DMD, Duchenne muscular dystrophy.

Generally, DMD-related LGE pattern, thus fibrosis, initially involves the subepicardial/mid-wall of the lateral/inferolateral wall, then it gets transmural and spreads to other myocardial segments [48, 49] (Fig. 3). LGE may appear even before 10 years of age in some cases while most of them show it after 15 years of age and its progressive myocardial spread has been correlated with increasing age and declining EF [50]. Like in DCM, myocardial fibrosis detected by LGE seems to have a prognostic role in the arrhythmic evaluation of DMD patients; a recent retrospective study including DMD patients showed a correlation between more extensive LGE and higher VA and SCD burden [17]. However, an established quantitative or semi-quantitative standardized LGE grading system is still lacking, while an LGE threshold to define patients at higher risk of arrhythmic events hasn’t been determined yet, thus reducing the current yield of this tool in clinical practice.

Exercise CMR, a powerful and yet still underused technique, has recently been proposed to unmask early signs of cardiomyopathy in DMD individuals without overt cardiac disease. In one study, higher end-systolic volumes indexed for fat-free body mass (ESViFFM) at rest and during exercise, and lower stroke volume and indexed cardiac output during exercise seemed to show abnormalities in left ventricular systolic function [51].

However, as stated before, ventilators are commonly used to assist DMD oxygenation failure all long the DMD evolution. Even if CMR-compatible ventilators exist, some CMR sequencies require apnea phases, thus being demanding for these patients. Therefore, CMR feasibility should be assessed, and a thorough patient selection appear to be essential to obtain high-quality information from CMR in this population.

Lastly, considerable research effort has focused on the relationship between molecular changing and imaging technology. One of the aims of that research was to achieve a better understanding of the interaction between genetics, individual pathobiology, specific biomarkers and the fibrosis found at CMR. DMD patients showed a higher LGE ratio compared to controls in one study [52], starting from that, a linkage between specific biomarkers reflecting gene expression and LGE has been searched. Results showed a correlation between the presence of specific miRNAs and LGE at CMR, strengthening the idea that genetics and multimodal imaging are strongly bonded [52]. Further proof comes from the genetic mutations and CMR findings: patients predicted to have the cysteine-rich domain, C-terminal domain (both the N-terminal actin-binding and cysteine-rich domains) and those with in-frame mutations in dystrophin gene had a decreased risk of developing LGE. This result highlights the importance of genotype-phenotype study in DMD to predict the extension of cardiac involvement and to set up a personalized therapy [53].

### 5.3 Electrophysiological Study, 3-Dimensional Electroanatomic Mapping and Catheter Ablation

**Fig. 4.**
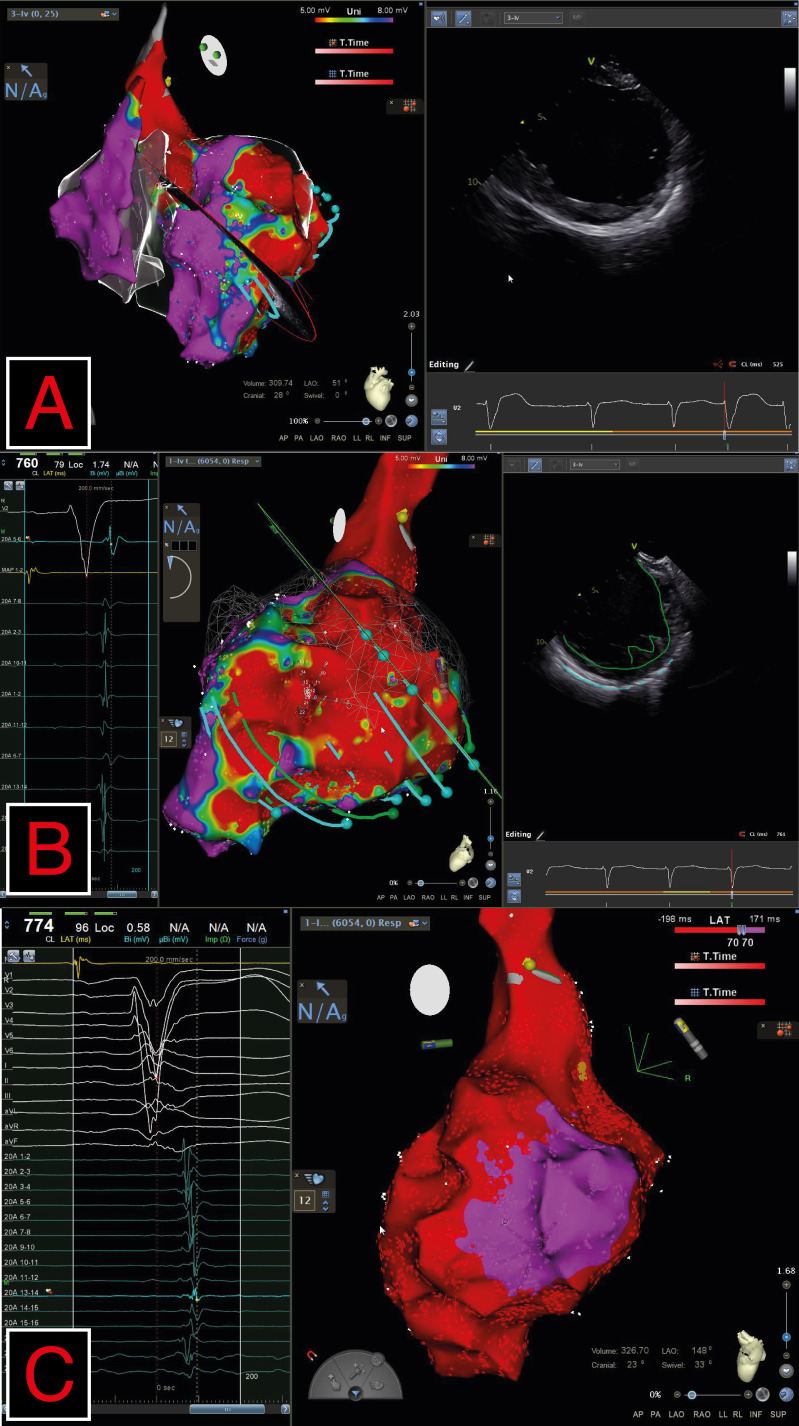
**Endocardial mapping of RV and LV by Carto3 system in a DMD patient and documented ventricular tachyarrhythmias**. (A) shows a large scar area (red area, voltage <5 mV) in the left lateral wall and the intracardiac echo view showing the inferolateral medial wall hyperechogenicity spreading from epicardial to midmyocardial layers. (B) shows a scar area from basal to apical regions. Green dots along intracardiac echo planes (green lines) of left lateral wall highlight late potentials detected by mapping in sinus rhythm as also indicated by ECG. (C) shows late potentials color timing map (on the right) with late potentials localized on left posterobasal wall. Late potentials after the end of QRS are recorded on the left side by CARTO EGM system. DMD, Duchenne muscular dystrophy; RV, right ventricular; LV, left ventricular; ECG, electrocardiogramL; LAT, local activation time; LAO, left anterior oblique; N/A, not applicable.

Electrophysiological study (EPS) and 3-Dimensional Electroanatomic mapping can detect specific affected myocardial areas possibly triggering arrhythmia onset (Fig. 4). Hidden intra-cardiac conduction disturbances, detected by EPS, predicts advanced block evolution and the need for pacemaker implantation in a considerable proportion of patients with NMDs. None of the patients with normal findings at EPS showed, instead, rhythm abnormalities during the follow-up [54]. To date, no evidence-based data supports the use of EPS in tachyarrhythmic risk stratification in DMD patients, either in the early or advanced phase of the disease. However, ESC recommendations on arrhythmic risk stratification in DCM have recently highlighted the emerging role of EPS with programmed electrical stimulation, for ICD implantation in primary prevention [8]. With regard to catheter ablation, it is recommended only in drug-refractory VTs [8].

### 5.4 Tele-Monitoring

In patients with unexplained syncope (or symptoms rising suspect of heart rhythm disturbances in general), loop recorder implantation and mobile/smart phone–based tele-monitoring may help in determining the burden of arrhythmias and their associations with concerning symptoms [32].

### 5.5 Multidisciplinary Approach

To provide the most patient-oriented strategy, a solid and collaborative multidisciplinary team of physicians (involving psychiatrists, neurologists, pneumologists, orthopedics, cardiologists, and infectious disease specialists) is essential [2, 7, 32]. Periodical multi-specialty team meetings seem the best way to identify critical elements of patients’ wellbeing allowing an active discussion about appropriateness and priority of interventions and the best therapeutic strategies for these patients. It should indeed be remarked that therapeutic targets are often difficult to assess in this population and they must be merged with those of their caregivers.

## 6. Ethical Considerations

Defining the arrhythmic and SCD risk in DMD patients entails several ethical questions. One may ask if it is it right to provide device implantation in a patient with an incurable disease, at high risk of peri- and intra-procedural complications, which is a commonly debated subject.

To date, we can state that ICD implantation (and device treatment in general) is a validated option to ameliorate clinical symptoms and prevent fatal arrhythmias and cardiac death in selected DMD patients. The lack of an evidence-based algorithm to identify patients with high arrhythmic risk still leaves many unresolved clinical, ethical, and practical questions, and highlights the urge for ICD implantation criteria standardization. To conclude, the process of decision-making should actively involve DMD patients, and an open and frank discussion is advocated to share the patient’s life expectations together with those of their families/caregivers.

## 7. Proposed Algorithm for SCD Prevention in DMD 

As previously stated, no DMD-specific criteria for primary prevention ICD implantation have yet been published . Below we present a proposal for a decision-making algorithm addressed to primary prevention ICD implantation in DMD patients. It is composed of three different categories: *non-cardiac* related, *cardiac*-related, and *patient*-related. Following the current SCD guidelines, our scheme integrates further elements for SCD risk stratification, overcoming the LVEF reduction paradigm (Central Figure).

**Central Figure.**
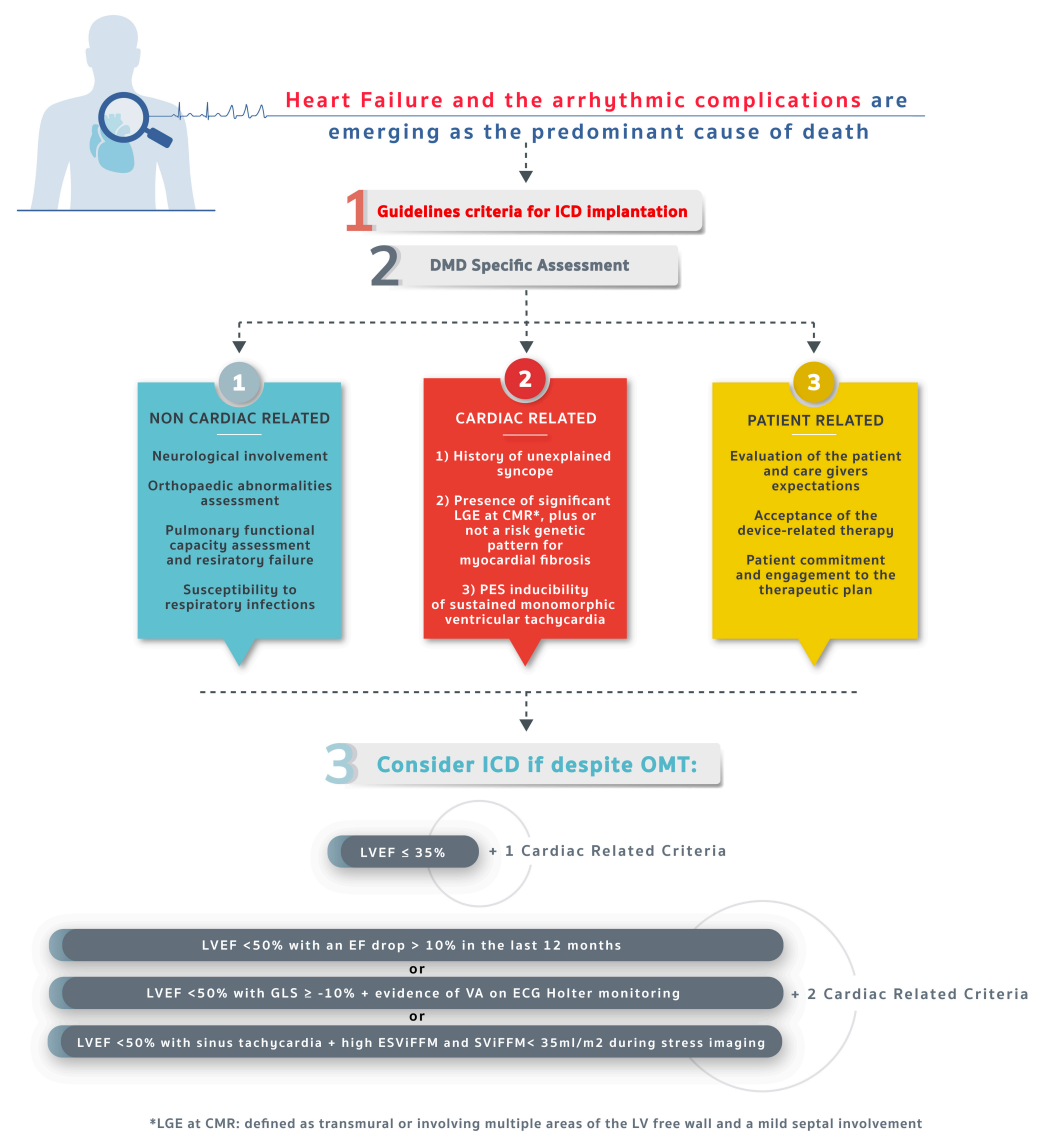
**Proposed decisional algorithm to ICD implantation in DMD patients**. Following a stepwise approach, integrating the EF value with information obtained from modern imaging modalities such as CMR and speckle tracking echocardiography, electrocardiographic and electrophysiological assessment, after specific disease-related and patient-related considerations, the ICD implantation can be considered case by case. (Some graphic elements used are under free license from freepik.com). DMD, Duchenne muscular dystrophy; ICD, implantable cardioverter-defibrillators; CMR, cardiac magnetic resonance; LGE, late gadolinium enhancement; OMT, optimal medical therapy; LVEF, left ventricular ejection fraction; EF, ejection fraction; VA, ventricular arrhythmia; ESViFFM, end-systolic volume indexed for fat-free body mass; SViFFM, stroke volume indexed for fat-free body mass; ECG, electrocardiogram; GLS, global longitudinal strain; LV, left ventricular; PES, programmed electrical stimulation.

Non-cardiac-related considerations include common comorbidities and medical aspects occurring in the natural history of the disease, which may result in implantation futility or harm. Neurological dysfunction, as well as musculoskeletal issues, must be considered, especially scoliosis and chest deformities that may lead to vein course abnormalities. Furthermore, special attention must be paid to concealed infections that need to be accurately ruled out, representing a contraindication to device implantation and an issue DMD patients are prone to. Specifically, lung infections due to diminished cough stimulus and skin wounds due to bedsores are commonplace in these patients [32].

Cardiac-related considerations include all the previously analyzed cardiovascular aspects that could have a role in optimizing ICD implantation choice. We propose a two-step algorithm. The first parameter that must be evaluated is EF as it defines groups with “high risk for arrhythmia”: (1) LVEF ≤35%; (2) LVEF <50% with an EF drop >10% in the last 12 months [55]; (3) EF <50% with GLS >/= –10% + evidence of VA at ECG Holter monitoring [45, 46]; (4) EF <50% with sinus tachycardia at rest + high ESViFFM and SViFFM <35 mL/mq during stress imaging [51]. As previously stated, growing evidence shows that EF alone may not be applied as a lone parameter to discuss ICD implantation, especially in non-ischemic cardiomyopathies. So, if the patient pertains to the LVEF ≤35% group a second high risk criteria must be fulfilled to put them in the “high risk” group; while for the other groups showing a declining EF, two additional high-risk criteria are demanded. Adjunctive high-risk criteria are unexplained syncope history, significant LGE at CMR (transmural or involving multiple areas of the LV free wall and a mild septal involvement) with additional risk genetic pattern for myocardial fibrosis and programmed electrical stimulation inducibility of sustained monomorphic ventricular tachycardia that may or may not be present.

Patient-related considerations include all the other aspects, especially those that are non-medical or not strictly clinically related. Firstly, the family background must be considered as well as the caregivers’ willingness to be responsible for the management of device controls and subsequent follow-up outpatient visits. Finally, the patient’s psychosocial type, their personal commitment and motivation must be assessed, evaluating their expectations about the implantation in terms of quality of life and life expectancy.

Taking it all into account, device implantation choice should be shared in a multidisciplinary team. The goal is to harmonize the clinical perspective (encompassing the global disease trajectory) with the patient and their caregivers’ needs so that a common pathway may be adopted.

## 8. Conclusions

Cardiovascular complications are emerging as the predominant cause of death in DMD patients. Fibro-fatty infiltration of the myocardium in DMD patients leads to scar tissue formation which contributes to ventricular dysfunction and might act as a substrate for malignant arrhythmia generation. To date, there is no specific DMD algorithm that may help in arrhythmogenic risk stratification. Likewise, traditional indications for ICD implantation are poorly investigated and not always applicable to this population. Furthermore, the decision to proceed with primary prevention ICD implantation bears ethical implications related to quality of life and its duration, particularly at the latest stages of the disease. We have therefore presented a new possible algorithm that may contribute towards ICD implantation choice in DMD patients to overcome these lack of recommendations.

To conclude, a collaborative approach between patients, caregivers, and a multidisciplinary team is warranted for this vulnerable patient population. A multidisciplinary clinical work-up that eases implantation decision choice should integrate multi-source elements, from CMR and speckle tracking echocardiography to electrocardiographic and electrophysiological assessments, always prioritizing patients’ and/or their caregivers’ preferences.

## 9. Perspectives: Bullet Points

• Cardiomyopathy is emerging as the main cause of death in DMD patients.

• An integrated evaluation could help form a tailored choice to individual patients.

• Cardiac parameters derived from modern imaging modalities and invasive studies together with a multidisciplinary clinical evaluation can positively impact on cardiac prognosis for DMD patients.
